# Early adjunctive methylene blue in patients with septic shock: a randomized controlled trial

**DOI:** 10.1186/s13054-023-04397-7

**Published:** 2023-03-13

**Authors:** Miguel Ibarra-Estrada, Eduardo Kattan, Pavel Aguilera-González, Laura Sandoval-Plascencia, Uriel Rico-Jauregui, Carlos A. Gómez-Partida, Iris X. Ortiz-Macías, José A. López-Pulgarín, Quetzalcóatl Chávez-Peña, Julio C. Mijangos-Méndez, Guadalupe Aguirre-Avalos, Glenn Hernández

**Affiliations:** 1 Unidad de Terapia Intensiva, Hospital Civil Fray Antonio Alcalde, Universidad de Guadalajara, Coronel Calderón 777, El Retiro, Guadalajara, Jalisco Mexico; 2 Instituto Jalisciense de Cancerología, Guadalajara, Jalisco Mexico; 3 The Latin American Intensive Care Network (LIVEN), Guadalajara, Mexico; 4 Departamento de Medicina Intensiva, Facultad de Medicina, Pontificia Universidad Católica de Chile, Santiago, Chile; 5 Servicio de Pediatría, Hospital Civil “Juan I. Menchaca”, Guadalajara, Jalisco Mexico

**Keywords:** Methylene blue, Randomized controlled trial, Septic shock, Norepinephrine, Vasopressin, Catecholamine sparing

## Abstract

**Purpose:**

Methylene blue (MB) has been tested as a rescue therapy for patients with refractory septic shock. However, there is a lack of evidence on MB as an adjuvant therapy, its’ optimal timing, dosing and safety profile. We aimed to assess whether early adjunctive MB can reduce time to vasopressor discontinuation in patients with septic shock.

**Methods:**

In this single-center randomized controlled trial, we assigned patients with septic shock according to Sepsis-3 criteria to MB or placebo. Primary outcome was time to vasopressor discontinuation at 28 days. Secondary outcomes included vasopressor-free days at 28 days, days on mechanical ventilator, length of stay in ICU and hospital, and mortality at 28 days.

**Results:**

Among 91 randomized patients, forty-five were assigned to MB and 46 to placebo. The MB group had a shorter time to vasopressor discontinuation (69 h [IQR 59–83] vs 94 h [IQR 74–141]; *p* < 0.001), one more day of vasopressor-free days at day 28 (*p* = 0.008), a shorter ICU length of stay by 1.5 days (*p* = 0.039) and shorter hospital length of stay by 2.7 days (*p* = 0.027) compared to patients in the control group. Days on mechanical ventilator and mortality were similar. There were no serious adverse effects related to MB administration.

**Conclusion:**

In patients with septic shock, MB initiated within 24 h reduced time to vasopressor discontinuation and increased vasopressor-free days at 28 days. It also reduced length of stay in ICU and hospital without adverse effects. Our study supports further research regarding MB in larger randomized clinical trials.

*Trial registration* ClinicalTrials.gov registration number NCT04446871, June 25, 2020, retrospectively registered.

## Background

Sepsis is a host’s dysregulated response to an infection, characterized by endothelial dysfunction leading to increased vascular permeability, abnormal nitric oxide (NO) metabolism, vasodilation, among other systemic derangements [1]. Along with antibiotics, the Surviving Sepsis Campaign (SSC) Guidelines recommend early fluid resuscitation as a cornerstone of management [2]; however, only half of the patients respond to fluid challenges [3] and their hemodynamic effects are transient, sometimes lasting as short as 10 min [4]; so, vasopressors are needed to improve organ perfusion. Norepinephrine is the first-choice [2], but high doses increase the risk of adverse effects such as tachyarrhythmia, myocardial dysfunction, peripheral ischemia, and even immunosuppression [5, 6]. Therefore, a combination of agents targeting different systems involved in blood pressure regulation and endothelial function has been recently proposed [7–9]. This “multimodal strategy” could help to restore tissue perfusion while decreasing the potential toxicity of single agents [10, 11].

Methylene blue (MB) is a specific inhibitor of the inducible nitric oxide synthase (iNOS) and its downstream enzyme soluble guanylate cyclase (sGC). Through its indirect pressor effects, it has been shown to restore vasoregulation in conditions of NO upregulation [12]. However, most of the clinical research has been performed in vasoplegia following cardiopulmonary bypass (CPB), another form of vasodilatory shock similar to sepsis [13]. Despite promising results of two small randomized controlled trials (RCT) with short follow-up (≤ 48 h) performed in patients with septic shock two decades ago [14, 15], the momentum to further research was shortly lost.

Based on large datasets, it is increasingly recognized that a higher exposure to catecholamine vasopressors is associated with an increased risk of multiple organ failure and death in septic shock [8, 16, 17]; thus, the time of norepinephrine requirement is a justified intermediate patient-centered outcome [18] in order to pave the way for adding catecholamine-sparing agents to a multimodal strategy [7, 19]. We designed this RCT to assess if early adjunctive MB administration could reduce the time to vasopressor discontinuation in patients with septic shock, as compared to placebo.

## Materials and methods

### Trial design and oversight

We conducted an investigator-initiated, parallel, double blinded, randomized controlled trial at an academic reference center in Mexico, in a medical-surgical intensive care unit (ICU). The study was approved by the institutional review board “Comité de Ética en Investigación Hospital Civil Fray Antonio Alcalde” (HCG/CEI-0252/17) and performed in accordance with the ethical standards as laid down in the 1964 Declaration of Helsinki and its later amendments. This trial was registered at clinicaltrials.gov (NCT04446871) after inclusion of 17 patients. Informed consent was obtained from all patients or decision makers.

### Patients

Patients aged ≥ 18 years with septic shock as defined by Sepsis-3 criteria (highly suspected or confirmed infection, requiring norepinephrine to maintain a MAP ≥ 65 mmHg, and serum lactate > 2 mmol/L after adequate fluid resuscitation) [20] were assessed for eligibility.

Exclusion criteria included > 24 h since initiation of norepinephrine, pregnancy, high probability of death within 48 h, concurrent hemorrhagic, obstructive or hypovolemic shock, pending damage control surgery, major burn injury, personal or familiar history of glucose-6-phosphate dehydrogenase deficiency, allergy to methylene blue, phenothiazines, or food dyes, recent intake (4-weeks) of selective serotonin re-uptake inhibitors, and refusal of the patient or decision maker to participate. During the beginning of coronavirus disease (COVID-19) pandemic, data safety monitoring board did not allow us to recruit patients with this diagnosis, due to the unknown pathophysiologic underpinnings and the uncertainties about response to MB.

### Randomization, intervention and measurements

After signing informed consent, patients were randomly assigned to receive MB using a predetermined randomization sequence prepared in sealed opaque envelopes. The sequence was generated by computer with a 1:1 allocation ratio, using permuted blocks with a size of 4. Critical care physicians were responsible for assignment of intervention. Patients, clinicians, investigators and outcome assessors were blinded to the treatment received.

Patients assigned to MB group received an intravenous (IV) infusion of 100 mg of MB in 500 ml of 0.9% sodium chloride solution over 6 h once daily for a total of 3 doses. Patients assigned to control group received the same dose of 500 ml of 0.9% sodium chloride without MB. In order to avoid visual identification of the infusion, all infusion bags and polyvinylchloride lines were prepared at central pharmacy with opaque envelopes.

In our unit, fluid resuscitation of septic shock is guided by dynamic tests for prediction of volume responsiveness before any IV fluid load; the most common methods in order of frequency are aortic velocity–time integral change after passive leg raising (cut-off 10%), arterial pulse pressure variation (cut-off 13%), tidal volume challenge (cut-off 3.5%), and respiratory variation of carotid peak flow velocity (cut-off 14%) [21]. We defined adequate fluid resuscitation as at least 500 ml bolus of balanced crystalloid followed by negative volume responsiveness by at least 2 different methods.

In patients of both groups, adjunctive vasopressin was initiated at a dose of 0.03 IU/min if norepinephrine dose reached ≥ 0.25 mcg/kg/min; evaluation of volume responsiveness was repeated at least 3 times each day as long as vasopressors were needed. Hydrocortisone at 200 mg/day dose by continuous infusion is also a standard in our unit, and it is withheld within 6-h after discontinuation of all vasopressors without taper [22]. Nurse-led vasopressor tapering protocol consisted of norepinephrine titrated at 15–20-min intervals to maintain mean arterial pressure (MAP) between 65 and 75 mmHg until complete discontinuation, and vasopressin was progressively withdrawn by 0.005 UI/min each hour only after complete discontinuation of norepinephrine.

Recorded information at randomization included demographic, ventilatory and laboratory data, including diagnosis of acute respiratory distress syndrome (ARDS) defined according to Berlin criteria [23]. Norepinephrine dose was recorded at randomization, immediately after each dose of intervention drug, at 24- and 48-h post-treatment, for a total of 4 days. Comprehensive multiorgan point-of-care ultrasound was performed at enrollment and as needed by critical care physicians with > 8 years of experience in critical care ultrasound, who are all certified trainers of the WINFOCUS (*World Interactive Network Focused on Critical Ultrasound*, https://www.winfocus.org/) international training unit. Ejection fraction was calculated by Teichholz’s method. Methemoglobin capillary saturation was continuously monitored along the intervention timeframe by pulse co-oximetry (Masimo Rainbow Set, Irvine, CA, USA) and maximum daily values were recorded.

### Outcomes

All patients were followed up until 28 days of enrollment, with time to vasopressor discontinuation as the primary outcome, defined as discontinuation of all vasopressors for at least 48 consecutive hours.

Secondary outcomes included vasopressor-free days at 28 days, all-cause mortality at 28 days, serum lactate levels, days on mechanical ventilator, length of stay in ICU and hospital; and the change in serum creatinine, bilirubin, aspartate/alanine aminotransferase, PaO_2_/F_I_O_2_ ratio and ejection fraction after intervention.

### Sample size

According to data of a previous study at our settings [22], with an expected mean vasopressor duration of 97 ± 69 h (median 83 h), and considering a decrease of 24 h as clinically relevant, the calculated sample size was 88 patients for the trial to provide a statistical power of 80%, and α-error of 0.05. Assuming a minimal attrition rate, we aimed to recruit 92 patients in total (46 per group).

### Statistical analysis

Numeric data are expressed as a percentage (%), using *x*^2^ or Fisher’s exact test for comparison as appropriate. According to Shapiro–Wilk test for normality, normally distributed data are expressed as means ± standard deviation, and skewed data are reported as medians with interquartile ranges 25th–75th (IQR). Comparison of continuous variables between groups was made with Student’s t or Mann–Whitney U test, as appropriate; repeated measures analysis of variance or Friedman’s test was used to compare variables at different points in time. A Kaplan–Meier curve was plotted for vasopressor discontinuation and analyzed with death as a competing risk event using Fine and Gray test with proportional hazards model. All *p* values were two-sided, and a value < 0.05 was considered as statistically significant. Outcomes were analyzed on an intention-to-treat basis. Statistical analysis and figures were performed with MedCalc (MedCalc Software Ltd Ostend, Belgium. Version 20.1), GraphPad Prism (version 9.3.1) and R version 4.2.2.

## Results

**Fig. 1:**
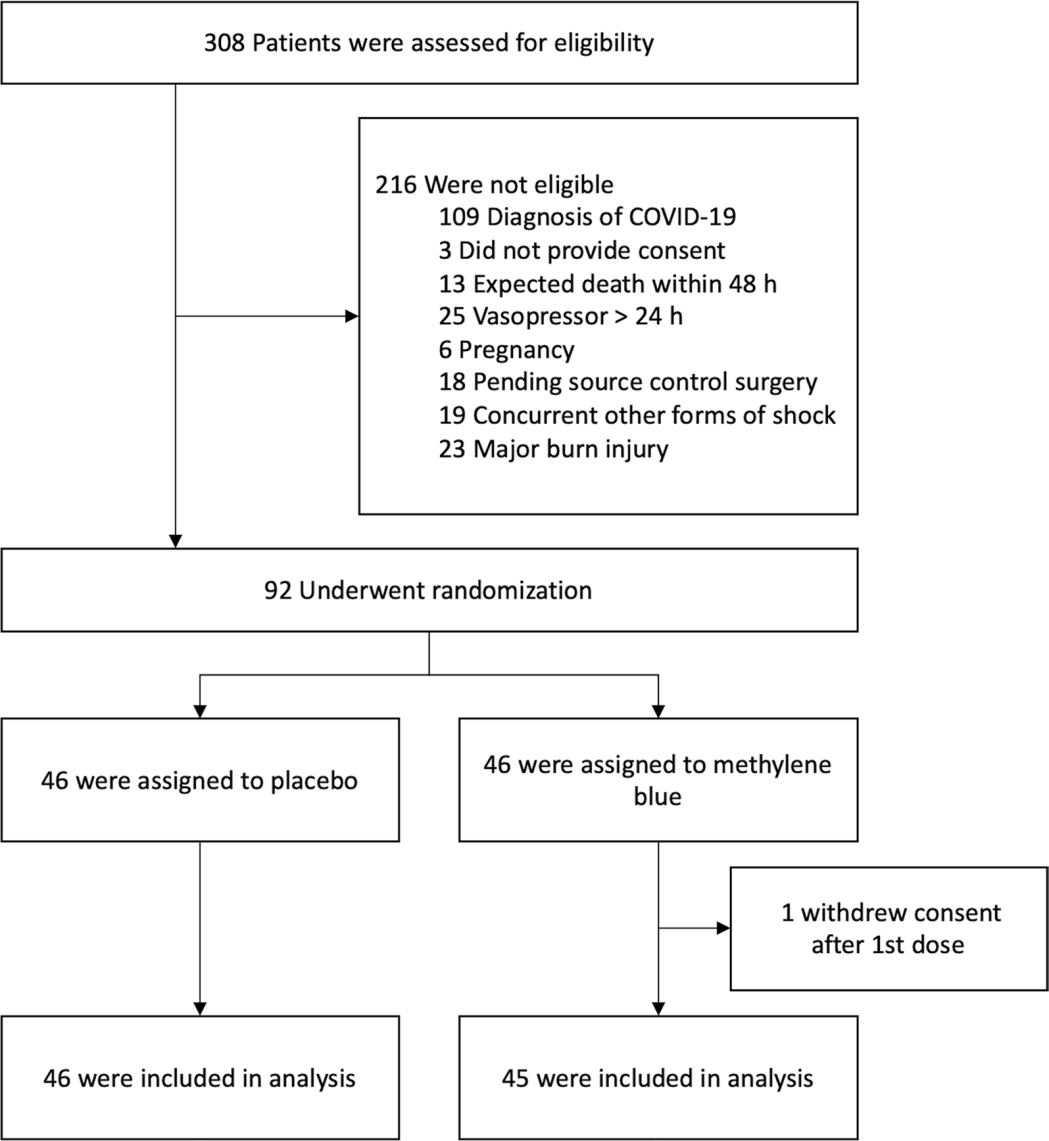
Flowchart of participants

From March 16, 2017, to July 30, 2022, 308 patients were assessed for eligibility, of whom 92 underwent randomization (Fig. 1). One patient of the MB group withdrew consent after the first dose of intervention; therefore, 91 patients were included in the analysis, with 45 patients assigned to MB and 46 to control group. One patient in the control group died before receiving the 3rd dose; all other patients received the complete scheme. All patients received hydrocortisone. Median age was 46 years (IQR 35–55), sixty percent were men, and 46% presented acute kidney injury (AKI). The most common sources of sepsis were pulmonary (49.5%) and intra-abdominal (38.5%). All patients received antimicrobial treatment within 3 h of septic shock diagnosis. Most patients were on mechanical ventilation and received the assigned intervention after initial 6-h from shock identification. These and other baseline characteristics were similar in both groups (Table 1). The daily weight dose of MB in the intervention group was 1.2 mg/kg (IQR 1.1–1.4). No other vasopressors (phenylephrine, angiotensin II, epinephrine, midodrine) or inotropes (milrinone, dobutamine) were used.

**Table 1:** Baseline characteristics according to allocated group

Characteristics	MB (*n* = 45)	Control (*n* = 46)
Age—years, median (IQR)	46 (38–54)	47 (31–60)
Female sex—no. (%)	18 (40)	18 (40)
Hypertension—no. (%)	17 (38)	18 (39)
Diabetes—no. (%)	19 (42)	17 (37)
Acute kidney injury—no. (%)	22 (49)	20 (44)
Infection source—no. (%)		
Pneumonia—no. (%)	22 (49)	23 (50)
Intra-abdominal—no. (%)	17 (38)	18 (39)
Urinary tract—no. (%)	4 (9)	4 (9)
Other—no. (%)	2 (4)	1 (2)
Shock diagnosis to intervention—hours, mean (SD)	8.3 ± 1.7	7.6 ± 2.3
Positive fluid response at enrollment— no. (%)	22 (49)	20 (44)
Fluid load from shock diagnosis to intervention—ml/kg, mean (SD)	24 ± 8.4	22 ± 9.6
Heart rate, mean (SD)	114 ± 9.9	115 ± 10.5
Mean arterial pressure—mmHg, mean (SD)	68 ± 4.5	67 ± 4.3
Norepinephrine dose—mcg/kg/min), median (IQR)	0.45 (0.27–0.68)	0.37 (0.20–0.58)
Vasopressin use—no. (%)	36 (80)	34 (74)
Serum lactate—mmol/L, median (IQR)	6.3 (4.8–7.4)	5.0 (2.9–7.5)
Mechanical ventilation—no. (%)	42 (93)	38 (82)
ARDS—no. (%)	36 (80)	32 (70)
PaO_2_/F_I_O_2_ ratio (SD)	190 ± 73	219 ± 79
PEEP—cmH_2_O, IQR	8 (6–8.5)	7 (6–9)
Serum creatinine—mg/dL, median (IQR)	1.5 (0.7–2.5)	1.5 (0.8–2.1)
Serum bilirubin—mg/dL, median (IQR)	1.4 (1.1–1.7)	1.3 (0.8–1.6)
AST—mg/dL, median (IQR)	113 (99–142)	107 (75–163)
ALT—mg/dL, median (IQR)	48 (41–78)	39 (30–79)
Ejection fraction—%, median (IQR)	61 (55–70)	58 (54–66)
SOFA, median (IQR)	10 (8–12)	10 (8–12)
APACHE II, mean (SD)	22.9 ± 4.4	22.4 ± 4.4

Plus–minus values are means ± standard deviation (SD); median with interquartile ranges (IQR) are in parentheses

*MB* methylene blue, *ARDS* acute respiratory distress syndrome, *PEEP* positive end-expiratory pressure, *AST* aspartate aminotransferase, *ALT* alanine aminotransferase, *APACHE* Acute Physiology and Chronic Health Evaluation

### Primary outcome

**Fig. 2:**
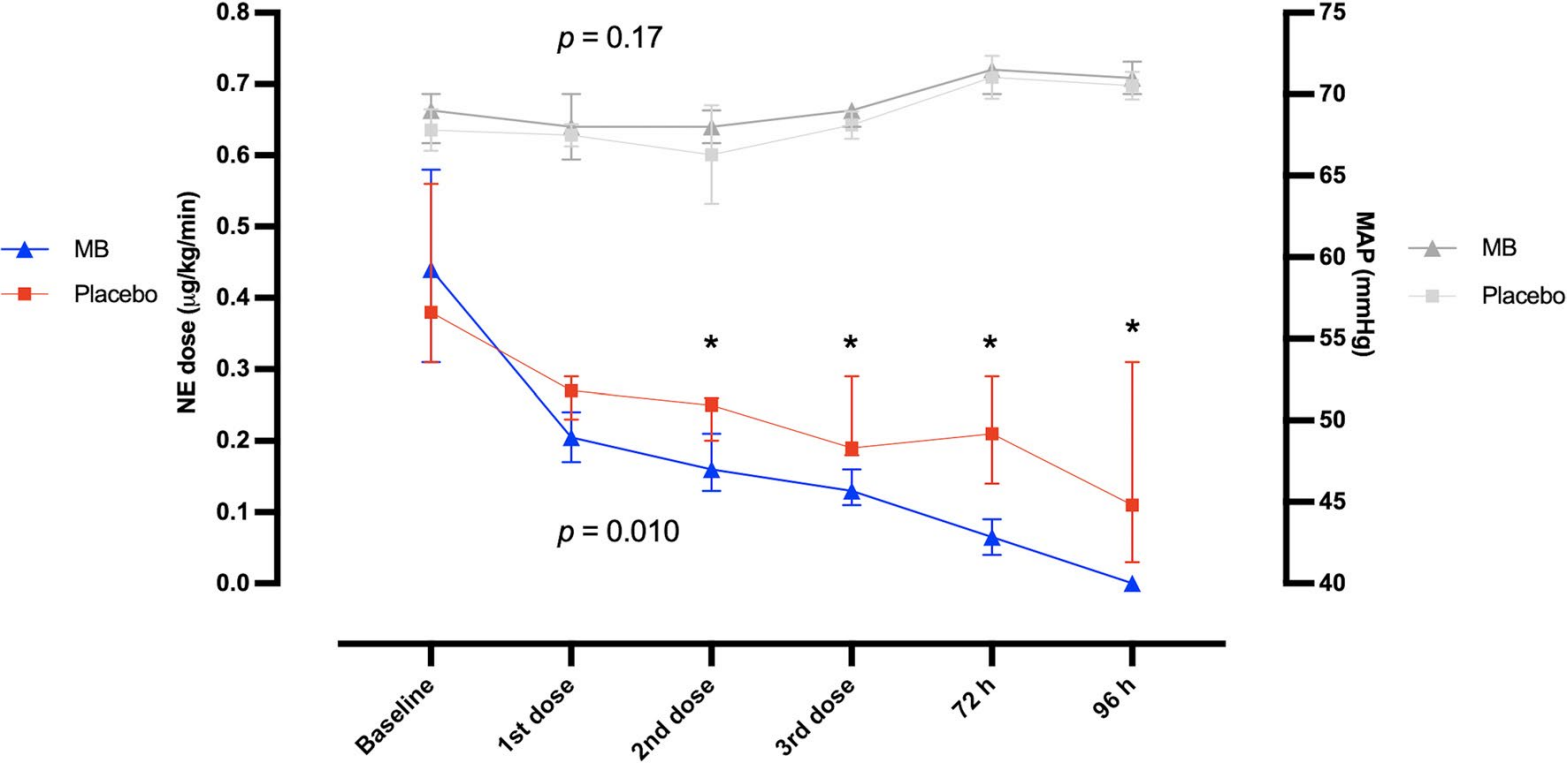
Trends of mean arterial pressure and norepinephrine requirement along the first 4 days after recruitment. *p* values reflect the between subjects (groups) comparison test. * *p* < 0.05 between groups

The time to vasopressor discontinuation was 69 h (IQR 59–83) in the MB group and 94 h (IQR 74–141) in the control group (median difference 29.4 [15.4–50.7]; *p* < 0.001). Norepinephrine dose requirement decreased more pronouncedly in the MB group as compared to placebo along the first 4 days (Fig. 2). A total of 5 of 45 (11%) patients in the MB group and 13 of 46 (28%) patients in the control group required re-initiation of NE within 48 h after discontinuation (*p* = 0.06).

### Secondary outcomes

**Fig. 3:**
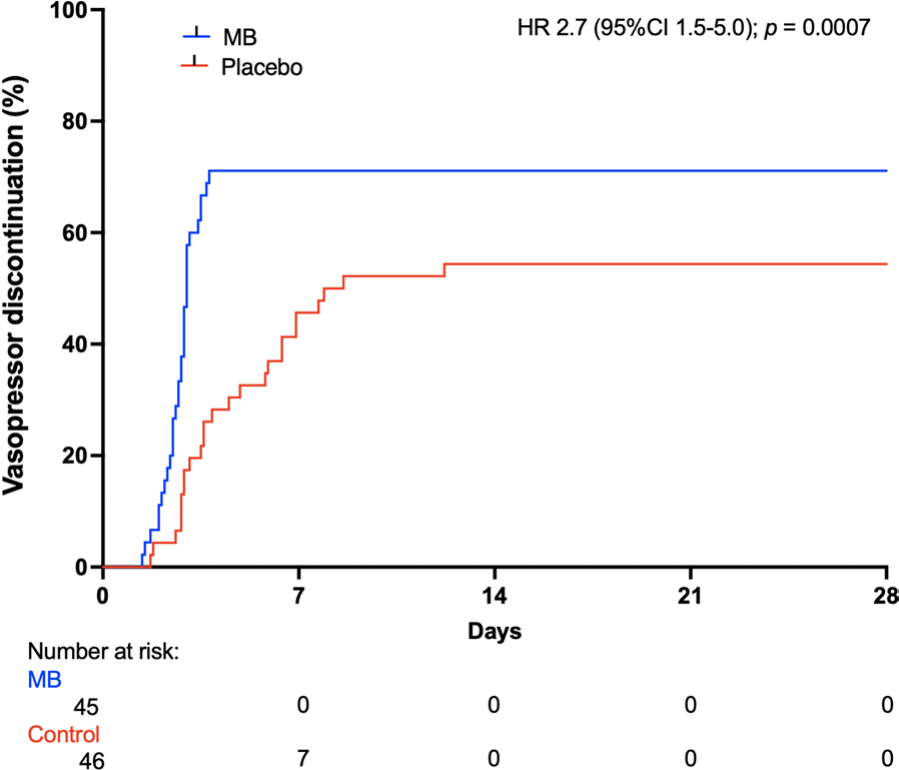
Kaplan–Meier plot of the cumulative incidence of vasopressor discontinuation. Adjusted hazard ratio with death as a competing risk analysis. *MB* methylene blue, *HR* hazard ratio

Patients in the MB group had 1.0 more days of vasopressor-free days at day 28 (*p* = 0.008). They had a lower cumulative fluid balance by 741 ml (CI_95_ 293–1188; *p* = 0.001), a shorter ICU length of stay by 1.5 days (CI_95_ 0.08–2.5; *p* = 0.039), and shorter hospital length of stay by 2.7 days (CI_95_ 0.3–4.6; *p* = 0.027) compared to patients in the control group. At proportional hazards analysis, we found a hazard ratio for shock reversal of 2.7 for patients in the MB group at 28 days (CI_95_ 1.5–5.0; *p* = 0.0007) (Fig. 3). Lactate levels within the first 3 days, days on mechanical ventilation and mortality at 28 days were similar (Table 2).

**Table 2:** Outcomes according to allocated group

Outcomes	MB (*n* = 45)	Control (*n* = 46)	Median difference (CI_95_)	*p*
Time to vasopressor discontinuation—hours, median (IQR)	69 (59–83)	94 (74–141)	29.4 (15.4–50.7)	< 0.001
Vasopressor-free days at 28 days, median (IQR)	23.9 (0.0–24.8)	19.5 (0.0–23.7)	1.0 (0–4.1)	0.008
Cumulative fluid balance at 4 days, mean (SD)	834 ± 1106	1575 ± 1040	741 (293–1188)	0.001
Days on mechanical ventilation, median (IQR)	4.2 (3.3–5.1)	5.1 (3.9–5.9)	0.6 (− 0.06–1.2)	0.075
Serum lactate—mmol/L, median (IQR)				
24 h	2.9 (2.2–3.7)	3.1 (2.6–6.0)	0.5 (0.0–1.4)	0.054
48 h	1.7 (1.4–3.4)	2.5 (1.1–3.3)	0.3 (− 0.5–0.9)	0.36
72 h	1.4 (1.0–2.1)	1.7 (0.5–3.1)	0.0 (− 0.06–0.7)	0.98
ICU length of stay—days, median (IQR)	6.6 (4.8–7.6)	7.9 (5.0–10.0)	1.5 (0.08–2.5)	0.039
Hospital length of stay—days, median (IQR)	9.0 (6.3–9.3)	10.5 (6.1–14.0)	2.7 (0.3–4.6)	0.027

			Relative Risk (CI_95_)	
Mortality at 28 days—no. (%)	15/45 (33)	21/46 (46)	0.76 (0.55–1.05)	0.23

Median with interquartile ranges are in parentheses

*MB* Methylene blue, *CI* confidence interval, *ICU* intensive care unit

### Adverse effects

**Fig. 4:**
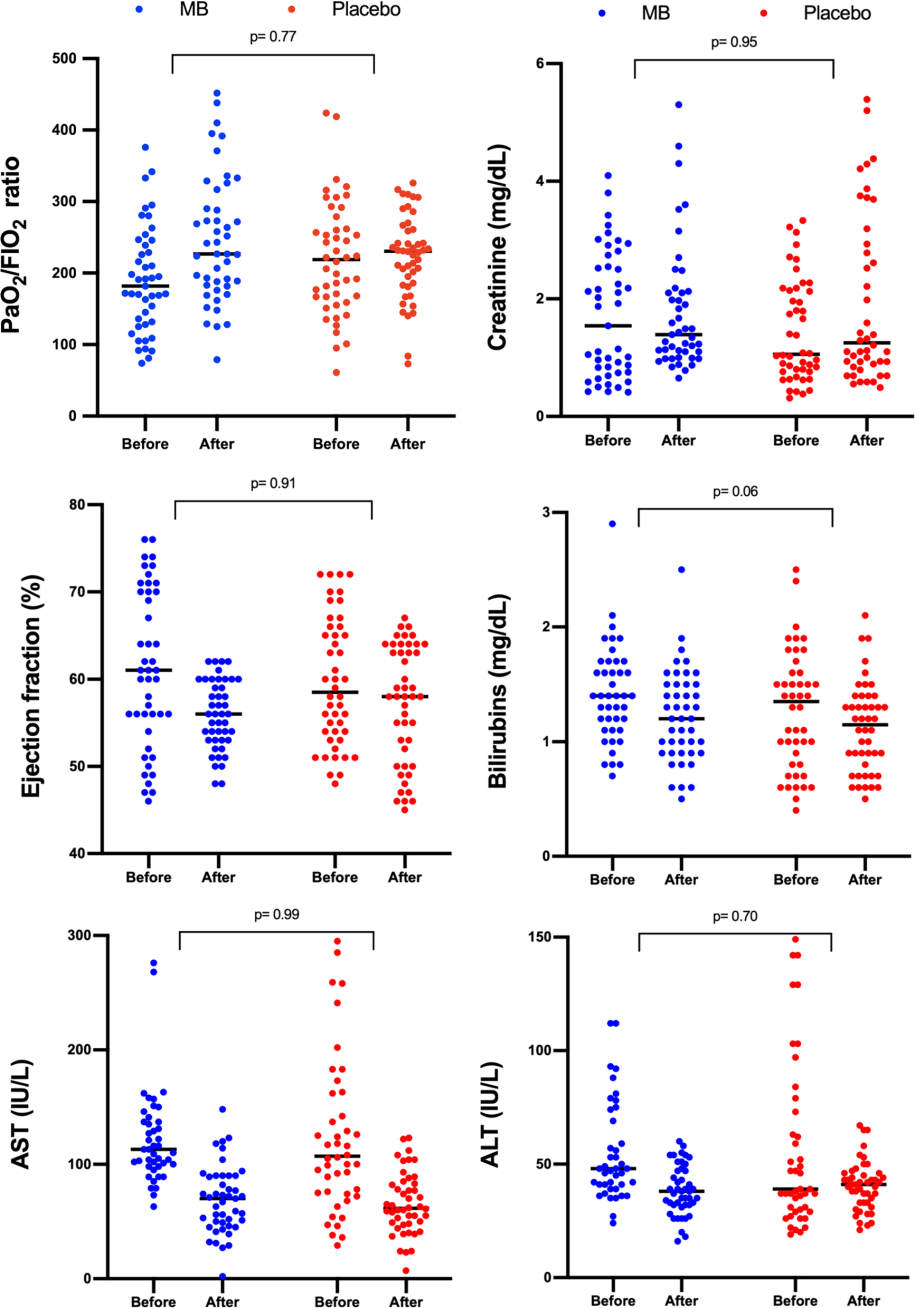
Comparison of the change in monitoring values before initiation and after the last dose of intervention. *p* values reflect the between subjects (groups) comparison test. *MB* methylene blue, *AST* aspartate aminotransferase, *ALT* alanine aminotransferase

The most common adverse effect was green–blue discoloration of urine in 42 of 45 (93%) patients in the MB group. Maximum methemoglobin saturation was significantly higher in patients of the MB group (2.9% [IQR 2.2–3.3] vs 0.5% [IQR 0.4–0.7]; *p* < 0.001). Regarding other potential adverse effects, the change in ejection fraction, PaO_2_/F_I_O_2_, serum creatinine, bilirubins and liver aminotransferases were not different between groups after the intervention (Fig. 4).

## Discussion

In this single-center RCT, we found that adjunctive MB administered within 24 h of septic shock diagnosis reduced time to vasopressor discontinuation, and importantly, no severe adverse effects were detected. The clinical implications of our results might shift the current understanding of MB as a rescue therapy [24] to an adjunctive one at earlier stages of the disease. Due to its safety profile, wider availability and lower cost than other catecholamine-sparing agents [25, 26], MB could emerge as a viable therapy within a multimodal strategy to maintain MAP and improve tissue perfusion, while decreasing the risk of high-dose vasopressors [9, 27–29].

To our knowledge, this is the largest RCT comparing MB vs placebo in patients with septic shock. In the study by Kirov et al. in 2001, they randomized 20 patients with septic shock to placebo or a bolus injection of 2 mg/kg of MB, followed by a continuous 4-h infusion to a total dose of 5.75 mg/kg, and followed patients up to 24 h. Although more patients died in the control group (7 vs 3) and duration of vasopressor support was shorter in MB group (71 h vs 93 h), these differences were not significant [14]. In 2002, Memis et al. randomized 30 patients with septic shock to placebo or an infusion of MB at 3 mg/kg over 6 h, with a follow-up of 48 h. They found no differences in cytokine levels or any other relevant outcome; nonetheless, they reported a significant but transient increase in MAP in patients of MB group. Shortly after publication of these 2 promising studies, a large phase III multicenter RCT which included 797 patients with septic shock was published. López et al. randomized patients to placebo or the NOS inhibitor *N*(G)-methyl-L-arginine hydrochloride (546C88). The study was terminated early due to a significant increase by 10% in mortality of cardiovascular cause at 28 days [30]. This seems to have halted the enthusiasm in research about NO inhibition; however, it is important to note that this molecule is non-selective, inhibiting both inducible and constitutive isoforms of NOS (unlike MB which selectively inhibits the inducible form). Sparing the constitutive isoform is important to maintain homeostasis even in sepsis; for instance, to improve microcirculatory flow, increase blood flow to ischemic areas, scavenge oxygen-free radicals, and enhance microbial killing by macrophages [31]. It has been postulated that a global inhibition of NO is associated with detrimental effects in sepsis that can result in accelerated organ damage [32]; thus, a more downstream inhibition within the NO cascade as provided by MB could be a better approach. In line with these assumptions, MB was associated with a reduction in mortality in a recent systematic review and meta-analysis pooling observational and RCT studies [33].

The most common use of MB for septic shock is reported as single infusions due to extrapolation from the predominant literature on CPB [14, 15, 34–39]; but unlike sepsis, the systemic inflammatory insult of CPB which occurs in up to 50% of the patients is limited to a few hours [13]. By contrast, the duration of the inflammatory insult in septic shock is less predictable, and we know that the expected duration of vasopressor requirement is of 2–3 days even in optimal conditions as in recent clinical trials, [40]. This is the reason we decided to administer MB in repeated doses. Considering that NE requirement was significantly lower in MB than in control group only after the second dose (Fig. 2), we believe our positive findings were in part due to this approach, which provided a longer timeframe of iNOS and sGC inhibition. Data from a recent large retrospective study support this assumption; Sari-Yavuz et al. studied different modes of administration of MB in critically-ill patients with shock, and they reported that the length of inhibition of the NO pathway could influence the response to MB, rather than the cumulative dose [41].

Regarding potential adverse effects, two prior small non-randomized studies suggested that MB could induce worsening of oxygenation due to pulmonary vasoconstriction. Gachot et al. reported a significant decrease of PaO_2_/F_I_O_2_ ratio from 229 to 162 after a 3 mg/kg bolus of MB over 10 min [35], and Weingartner et al. found a decrease of PaO_2_/F_I_O_2_ ratio from 168 to 132 after a dose of 4 mg/kg over 4 h [38]; however, these detrimental effects were transient and not confirmed by other RCTs including ours, as most of our patients had ARDS diagnosis and the change in PaO_2_/F_I_O_2_ ratio and days of mechanical ventilation were similar between groups after treatment. Moreover, it is known that unlike the beneficial effects, the toxic profile of MB is dose related, so the rate/dose used in those non-randomized studies could have been unsafe. A dose of 1 mg/kg has been shown to be enough to improve MAP, left ventricular function and tissue oxygenation in human septic shock, while ≥ 7 mg/kg might compromise splanchnic perfusion [42] and adverse effects are rarely present under 2 mg/kg [43]. Besides, continuous prolonged infusion of MB may lead to higher cumulative doses, which might be toxic and result in methemoglobinemia [15, 42]; this is the pragmatic reason why we decided to administer a fixed dose of 100 mg vials (available presentation at our institution) assuming that most patients would receive at least 1 mg/kg while avoiding high cumulative doses. This schedule resulted in a total cumulative dose of 3.6 mg/kg over 54 h, which was sufficient to reduce vasopressor support with no detrimental effects in pulmonary, kidney, cardiac or liver function. Although methemoglobin levels were significantly higher in patients of MB, this elevation was far from the clinically relevant threshold of 10% [44].

### Limitations

Some limitations should be acknowledged. First, this was a single-center study. As a one of the largest reference centers in Mexico, it is common that our patients have a relatively short ICU/hospital stance compared to other larger multicenter trials [40]; as long as there is no need for strictly needed care, persistent organ failure or pending surgery/procedure, patients are discharged as rapidly as possible and followed up through outpatient visits. Thus, a different effect of MB on length of stay in other settings cannot be ruled out. Second, most patients were managed in other departments at the moment of septic shock identification before admission to ICU, the lack of trained healthcare staff and resources (ultrasound, invasive monitoring) might have led to sub-optimal resuscitation and worse patients’ status, as shown by the relatively higher doses of norepinephrine compared to other larger RCTs of patients with septic shock [45]. Therefore, although the time from enrollment to intervention was negligible, an improved response to MB at an earlier phase of the disease cannot be ruled out, as it has been suggested that MB could be more effective within an early “window of opportunity” [24]. Third, this trial took many years to complete as the COVID-19 pandemic slowed down the recruitment rate, and the SSC guidelines were updated while this study was still ongoing [2]. However, it is worth to note that the changes were few, and the resuscitation protocol in our study was still consistent with the new guidelines. For instance, we did not aim for a dose of 30 ml/kg of IV crystalloids at initial resuscitation, and this recommendation was downgraded; we also used IV corticosteroids in all patients while this recommendation was upgraded [2]. Fourth, we did not measure cytokine or nitrate/nitrite serum levels to confirm the mechanism of the effects of MB, but this should not distract from the pragmatic finding that MB reduced vasopressor duration, and ICU and hospital length of stay. Fifth, as we excluded patients with COVID-19, the benefit of MB still needs to be confirmed for this subgroup in future RCTs. Sixth, despite the effort to maintain blinding regarding allocation, the high incidence of urine discoloration and continuous assessment of methemoglobin levels through co-oximetry could have led to identification of group assignment, therefore, a biased adjustment of vasopressors by clinicians cannot be ruled-out. Nonetheless, the similar MAP during the first 4 days (Fig. 2) and less fluid administration suggests that if present, this bias was minimal. Lastly, the difference in mortality trends between groups should be interpreted cautiously, as this study was underpowered to draw any conclusion on this outcome, and larger studies should confirm these results.

## Conclusions

In conclusion, early adjunctive MB administration reduced vasopressor duration, cumulative fluid balance, and ICU and hospital length of stay among patients with septic shock, as compared with placebo. There were no severe adverse effects related to its use. Our results support the continuous research of MB as an early adjunctive therapy in patients with septic shock to confirm the potential benefit in larger multicenter randomized clinical trials.

## Data Availability

After publication, de-identified data will be available for sharing to researchers who provide a methodologically sound and ethically approved proposal, after approval of data sharing agreement and for research purposes only.

## Abbreviations

SCC: Surviving sepsis campaign
MB: Methylene blue
sGC: Soluble guanylate cyclase
iNOS: Inducible nitric oxide synthase
NO: Nitric oxide
CPB: Cardiopulmonary bypass
RCT: Randomized controlled trial
ICU: Intensive care unit
IV: Intravenous
COVID-19: Coronavirus disease
MAP: Mean arterial pressure
APACHE: Acute physiology and chronic health evaluation
WINFOCUS: World interactive network focused on critical ultrasound
IQR: Interquartile range
ROC: Receiver operating characteristic
AKI: Acute kidney injury
ARDS: Acute respiratory distress syndrome
PEEP: Positive end-expiratory pressure
CI_95_: Confidence interval
HR: Hazard ratio
